# Symmetry Properties of Mixed and Heat Photo-Assisted Noise in the Quantum Hall Regime

**DOI:** 10.3390/e21080730

**Published:** 2019-07-25

**Authors:** Flavio Ronetti, Matteo Acciai, Dario Ferraro, Jérôme Rech, Thibaut Jonckheere, Thierry Martin, Maura Sassetti

**Affiliations:** 1 Dipartimento di Fisica, Università di Genova, Via Dodecaneso 33, 16146 Genova, Italy; 2 Aix Marseille Univ, Université de Toulon, CNRS, CPT, Marseille, France; 3 SPIN-CNR, Via Dodecaneso 33, 16146 Genova, Italy

**Keywords:** electron quantum optics, photo-assisted noise, charge and heat fluctuations

## Abstract

We investigate the photo-assisted charge-heat mixed noise and the heat noise generated by periodic drives in Quantum Hall states belonging to the Laughlin sequence. Fluctuations of the charge and heat currents are due to weak backscattering induced in a quantum point contact geometry and are evaluated at the lowest order in the tunneling amplitude. Focusing on the cases of a cosine and Lorentzian periodic drive, we show that the different symmetries of the photo-assisted tunneling amplitudes strongly affect the overall profile of these quantities as a function of the AC and DC voltage contributions, which can be tuned independently in experiments.

## 1. Introduction

The possibility to generate, manipulate and detect single- to few-electron excitations coherently propagating in mesoscopic quantum conductors represents one of the main tasks in condensed matter. Theoretical and experimental studies in this direction culminated with the development of the new field of electron quantum optics (EQO) [1,2,3]. In this context, a remarkable experimental effort has been devoted to the realization of on-demand sources of electronic wave-packets. With this purpose, two main techniques have been elaborated. The first relies on a driven quantum dot [4,5], which plays the role of a mesoscopic capacitor [6,7,8,9], tunnel coupled to a Quantum Hall edge channel. This kind of device allows the periodic injection of an electron and a hole along the edge channels of the Hall bar. An alternative approach involves the generation of purely electronic wave-packets by applying a properly designed train of Lorentzian voltage pulses in time to a quantum conductor [10,11,12,13]. The experimental realization of these excitations, dubbed Levitons [14], renewed the interest in the so-called photo-assisted quantum transport, namely the study of the current generated by periodic drives and its fluctuations [15,16].

Even if experimentally challenging, the implementation of EQO in strongly correlated systems, such as Fractional Quantum Hall (FQH) edge channels, could open new and interesting perspectives in the field. In particular, peculiar features like the unexpected robustness of Levitons [17,18] and the crystallization of multiple-electronic wave-packets in the time domain [19,20] have been recently theoretically predicted. The simplest possible set-up to access this new physics requires a periodic train of voltage pulses applied to one of the terminals of a Hall bar in a Quantum Point Contact (QPC) geometry. Here, excitations incoming from the lead are partitioned at the QPC, in an electronic equivalent of the Hanbury–Brown and Twiss optical interferometer [21,22].

Notwithstanding the control of charge transport at the individual excitation level still presenting interesting and fascinating open problems, electric charge is not the only relevant degree of freedom we can look at in the framework of EQO. Indeed, electronic wave-packets also carry energy in a coherent way [23]. This observation is of particular importance in view of the progressive miniaturization of electronic devices, which makes the problem of heat transfer at the nanoscale extremely timely [24], as demonstrated by recent progress in the field of quantum thermodynamics [25]. In this framework, new intriguing challenges are posed by the need of properly transposing concepts such as energy harvesting [26,27,28,29,30,31,32,33], transport [34,35,36,37,38,39,40] and exchange [41,42,43] at the mesoscopic scale.

In this direction, new studies in the EQO domain have also been triggered. For instance, heat current was identified as a useful resource for the full reconstruction of single-electron wave-packets [44]. Intriguingly, also mixed heat-charge correlations [45,46,47] and heat current noise [48,49] were investigated in the case of single-electron sources with Levitons emerging as robust excitations also for what it concerns heat transport [50]. Despite a direct observation of heat current fluctuations is still lacking, an experimental protocol has been recently proposed in order to extract this quantity from temperature fluctuations [51].

In this paper, we evaluate the photo-assisted mixed and heat noise for FQH states in the Laughlin sequence [52]. Considering the QPC in a weak backscattering regime, we can confine our calculation to the lowest order in the tunneling. The aim will be to investigate the behavior of these quantities by independently tuning the AC and DC components of the voltage drive in the same spirit of what was done for the photo-assisted shot noise generated by electrical current [16,53].

The paper is organized as follows. In Section 2, we illustrate the model used to describe FQH edge channels coupled to a time dependent voltage. The charge current is evaluated at the lowest perturbative order in Section 3, while the corresponding expression for the heat current is derived in Section 4. In Section 5 we consider the charge and heat current fluctuations in terms of mixed and heat noise. Section 6 is devoted to the analysis of the symmetries of the discussed quantities as a function of the dc and ac contribution to the voltages. Finally, in Section 7 we draw our conclusions.

## 2. Model

**Figure 1:**
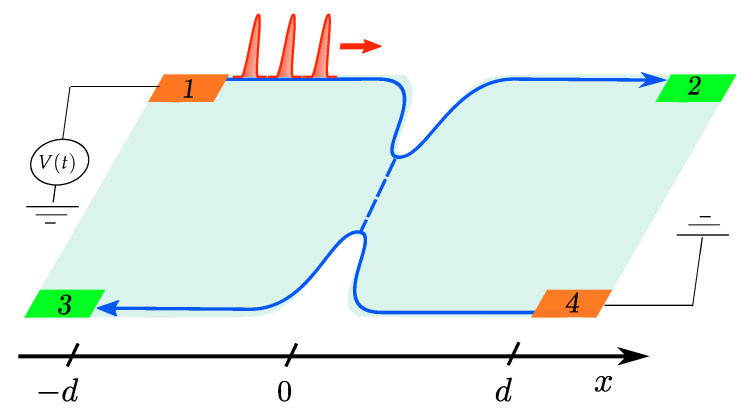
Four-terminal setup for an FQH state in the QPC geometry. Contact 1 is driven by a time dependent voltage V(t) and used as input terminal, contact 4 is grounded, while contacts 2 and 3 are the output terminals where currents and noises are measured. FQH: Fractional Quantum Hall; QPC: Quantum Point Contact.

We consider a four-terminal FQH bar in the presence of a QPC (see Figure 1). For a quantum Hall state with filling factor ν in the Laughlin sequence ν=1/(2n+1) [52], with n∈N, a single chiral bosonic mode emerges at each edge of the sample [54]. The effective Hamiltonian for right- and left-moving edge states (indicated in the following by *R* and *L* respectively) reads

(1) $$H_{0}=\sum_{r=R,L}\frac{v}{4\pi }\int_{-\infty }^{+\infty }dx{\left[\partial_{x}\Phi_{r}\left(x\right)\right]}^{2},$$

with $\Phi_{R/L}$ bosonic operators propagating with velocity *v*, assumed equal for both chiralities, along the edges. Notice that, from now on, we will set ℏ=1 for notational convenience.

The coupling between the electron particle density $\rho_{R}\left(x\right)=\frac{\sqrt{\nu }}{2\pi }\partial_{x}\Phi_{R}\left(x\right)$ and the coupling with a generic time dependent voltage gate V(t) applied to terminal 1 is encoded by the Hamiltonian

(2) $$H_{g}=-e\int_{-\infty }^{+\infty }dx\;\Theta \left(-x-d\right)V\left(t\right)\rho_{R}\left(x\right).$$

Here, the step function Θ(−x−d) models a homogeneous contact which is extended with respect to the Hall sample. In the following, we will focus on a periodic voltage drive of the form

(3) $$V\left(t\right)=V_{dc}+V_{ac}\left(t\right),$$

with

(4) $$\frac{1}{T}\int_{0}^{T}dtV_{ac}\left(t\right)=0,$$

T being the period of the drive.

The time-evolution of the bosonic field $\Phi_{R}$, according to the Hamiltonian $H=H_{0}+H_{g}$, is given by [17,19]

(5) $$\Phi_{R}\left(x,t\right)=\phi_{R}\left(t-\frac{x}{v}\right)-e\sqrt{\nu }\int_{-\infty }^{t-\frac{x}{v}}dt^{\prime }V\left(t^{\prime }\right).$$

Here, $\phi_{R}$ denotes the field which evolves with respect to $H_{0}$ only. These characteristic chiral dynamics are a direct consequence of the linear dispersion of edge modes.

Finally, we allow the tunneling of excitations between the two edges by locally approaching them, creating a QPC at x=0. This process can be effectively described by introducing the tunneling Hamiltonian [55]

(6) $$\begin{array}{cc} H_{T} & =\Lambda \Psi_{R}^{†}\left(0\right)\Psi_{L}\left(0\right)+h.c. \end{array}$$

Here, $\Psi_{R/L}$ ($\Psi_{R/L}^{†}$) are the annihilation (creation) operators for quasi-particles with fractional charge $e^{*}=-e\nu$. The Hamiltonian in Equation (6) describes the dominating tunneling process in the weak-backscattering regime [56]. In this regime, the tunneling Hamiltonian $H_{T}$ can be treated as a small perturbation with respect to *H*. As a consequence, the time evolution of quantum operators can be constructed in terms of a perturbative series in the tunneling amplitude Λ.

## 3. Charge Current

Charge current operators for right- and left-moving modes can be defined by means of the continuity equations of densities $\rho_{R/L}\left(x,t\right)$, namely

(7) $$-e\partial_{t}\rho_{R/L}\left(x,t\right)+\partial_{x}J_{R/L}\left(x,t\right)=0.$$

Due to the fact that the propagation of the modes long the channel is chiral [19], one finds

(8) $$J_{R/L}\left(x,t\right)=\mp ev\rho_{R/L}\left(x-vt\right),$$

where $\rho_{R/L}$ are the chiral density operators evolving in time according to the total Hamiltonian $H^{\prime }=H_{0}+H_{g}+H_{T}$.

Starting from the definition of the chiral current operator, we can define the operators for charge current entering reservoirs 2 and 3 as

(9) $$J_{2/3}\left(t\right)=J_{R/L}\left(\pm d,t\right),$$

where we recall that the interfaces between edge states and contacts 2 and 3 are placed at x=±d, respectively (see Figure 1). The expansion of $J_{2/3}$ in powers of Λ is given by

(10) $$J_{2/3}\left(t\right)=J_{2/3}^{\left(0\right)}\left(t\right)+J_{2/3}^{\left(1\right)}\left(t\right)+J_{2/3}^{\left(2\right)}\left(t\right)+O\left(\Lambda^{3}\right),$$

with

(11) $$\begin{array}{c} J_{2/3}^{\left(0\right)}\left(t\right)=\frac{ev\sqrt{\nu }}{2\pi }{\left(\partial_{x}\Phi_{R/L}\left(x,t\right)\right)}_{x=\pm d}, \end{array}$$

(12) $$\begin{array}{c} J_{2/3}^{\left(1\right)}\left(t\right)=\pm i\Lambda e\nu \Psi_{R}^{†}\left(0,t-\frac{d}{v}\right)\Psi_{L}\left(0,t-\frac{d}{v}\right)+h.c., \end{array}$$

(13) $$\begin{array}{c} J_{2/3}^{\left(2\right)}\left(t\right)=\pm i\int_{-\infty }^{t-\frac{d}{v}}dt^{\prime \prime }\;\left[H_{T}\left(t^{\prime \prime }\right),+i\Lambda e\nu \Psi_{R}^{†}\left(0,t-\frac{d}{v}\right)\Psi_{L}\left(0,t-\frac{d}{v}\right)+h.c.\right]. \end{array}$$

Tunneling contributions entering reservoirs 2 and 3 are connected by the simple relation

(14) $$J_{2}^{\left(1/2\right)}\left(t\right)=-J_{3}^{\left(1/2\right)}\left(t\right).$$

The thermal average of current operators will be performed over the initial equilibrium condition, i.e., in the absence of driving voltages ($H_{g}$) and tunneling ($H_{T}$). It is worth noting that $\langle J_{2/3}^{\left(1\right)}\left(x,t\right)\rangle$ is zero because it involves a different number of annihilation or creation field operators. Therefore, the average values of charge current operators satisfy

(15) $$\langle J_{2/3}\left(t\right)\rangle =\langle J_{2/3}^{\left(0\right)}\left(t\right)\rangle +\langle J_{2/3}^{\left(2\right)}\left(t\right)\rangle +O\left(\Lambda^{3}\right),$$

with

(16) $$\begin{array}{c} \langle J_{2}^{\left(0\right)}\left(t\right)\rangle =\frac{e^{2}\nu }{2\pi }V\left(t-\frac{2d}{v}\right), \end{array}$$

(17) $$\begin{array}{c} \langle J_{3}^{\left(0\right)}\left(t\right)\rangle =0, \end{array}$$

(18) $$\begin{array}{c} \langle J_{2/3}^{\left(2\right)}\left(t\right)\rangle =\pm ie\nu v\int_{-\infty }^{t-\frac{d}{v}}dt^{\prime \prime }\;\langle \left[H_{T}\left(t^{\prime \prime }\right),+i\Lambda \Psi_{R}^{†}\left(d-vt,0\right)\Psi_{L}\left(d-vt,0\right)+h.c.\right]\rangle . \end{array}$$

The first term corresponds to the charge current emitted by the reservoir 1, which constitutes the main contribution to the detected currents in reservoir 2. In the absence of tunneling processes (Λ=0), these zero-order contributions would correspond to a periodic current generated by V(t). For this reason, the integral over one period T gives the total charge C transferred across the edge channel, namely

(19) $$C=\int_{-\frac{T}{2}}^{\frac{T}{2}}dt\langle J_{2}^{\left(0\right)}\left(t\right)\rangle =\frac{e^{2}\nu }{2\pi }\int_{-\frac{T}{2}}^{\frac{T}{2}}dtV\left(t-\frac{2d}{v}\right)=\frac{e^{2}\nu }{\omega }V_{dc}=-eq,$$

where we have introduced the drive frequency ω=2π/T of the voltage in Equation (3) and used the property in Equation (4).

Due to the QPC, some of the excitations emitted by contact 1 are backscattered and $\langle J_{2}^{\left(2\right)}\left(t\right)\rangle$ ($\langle J_{3}^{\left(2\right)}\left(t\right)\rangle$) represents the transmitted (reflected) current [55,57]. Due to the relation in Equation (14), backscattering currents are equal up to a sign, so that we can define

(20) $$J_{BS}\left(t\right)=\langle J_{3}^{\left(2\right)}\left(t\right)\rangle =-\langle J_{2}^{\left(2\right)}\left(t\right)\rangle .$$

According to Equation (18), this backscattering current can be written as

(21) $$\begin{array}{c} J_{BS}\left(t\right)=-e{\left|\Lambda \right|}^{2}\nu \int_{-\infty }^{t-\frac{d}{v}}dt^{\prime \prime }\;[G_{R}^{<}\left(t^{\prime \prime },t-\frac{d}{v}\right)G_{L}^{>}\left(t^{\prime \prime },t-\frac{d}{v}\right)-G_{L}^{<}\left(t^{\prime \prime },t-\frac{d}{v}\right)G_{R}^{>}\left(t^{\prime \prime },t-\frac{d}{v}\right)+\\ -G_{R}^{<}\left(t-\frac{d}{v},t^{\prime \prime }\right)G_{L}^{>}\left(t-\frac{d}{v},t^{\prime \prime }\right)+G_{L}^{<}\left(t-\frac{d}{v},t^{\prime \prime }\right)G_{R}^{>}\left(t-\frac{d}{v},t^{\prime \prime }\right)], \end{array}$$

where we introduced the quasi-particle Green’s functions

(22) $$\begin{array}{c} G_{R}^{<}\left(t^{\prime },t\right)=\langle \Psi_{R}{}^{†}\left(0,t^{\prime }\right)\Psi_{R}\left(0,t\right)\rangle =e^{-i\nu e\int_{t}^{t^{\prime }}d\tau V\left(\tau \right)}\langle \psi_{R}{}^{†}\left(0,t^{\prime }\right)\psi_{R}\left(0,t\right)\rangle , \end{array}$$

(23) $$\begin{array}{c} G_{L}^{<}\left(t^{\prime },t\right)=\langle \Psi_{L}{}^{†}\left(0,t^{\prime }\right)\Psi_{L}\left(0,t\right)\rangle =\langle \psi_{L}{}^{†}\left(0,t^{\prime }\right)\psi_{L}\left(0,t\right)\rangle , \end{array}$$

with $\psi_{R/L}$ the quasi-particle field operators evolving with respect to $H_{0}$ only. Analogous expressions can be derived for $G_{R}^{>}$ and $G_{L}^{>}$.

In terms of the bosonized picture [36,58,59,60], the quasi-particle field operators can be written as coherent states of the bosonic edge modes, namely

(24) $$\begin{array}{cc} \psi_{R}\left(x,t\right) & =\frac{F_{R}}{\sqrt{2\pi a}}e^{ik_{F}\left(x-vt\right)}e^{-i\sqrt{\nu }\phi_{R}\left(x,t\right)}, \end{array}$$

(25) $$\begin{array}{cc} \psi_{L}\left(x,t\right) & =\frac{F_{L}}{\sqrt{2\pi a}}e^{ik_{F}\left(x+vt\right)}e^{-i\sqrt{\nu }\phi_{L}\left(x,t\right)}, \end{array}$$

with $F_{R/L}$ Klein factors [59]. Therefore, the expressions for the Green’s functions in Equations (22) and (23) become

(26) $$\begin{array}{c} G_{R}^{<}\left(t^{\prime },t\right)=\langle \Psi_{R}{}^{†}\left(0,t^{\prime }\right)\Psi_{R}\left(0,t\right)\rangle =e^{-i\nu e\int_{t}^{t^{\prime }}d\tau V\left(\tau \right)}\frac{e^{ik_{F}v\left(t^{\prime }-t\right)}}{2\pi a}P_{\nu }\left(t-t^{\prime }\right), \end{array}$$

(27) $$\begin{array}{c} G_{L}^{<}\left(t^{\prime },t\right)=\langle \Psi_{L}{}^{†}\left(0,t^{\prime }\right)\Psi_{L}\left(0,t\right)\rangle =\frac{e^{-ik_{F}v\left(t^{\prime }-t\right)}}{2\pi a}P_{\nu }\left(t-t^{\prime }\right), \end{array}$$

where we introduced the function

(28) $$P_{\nu }\left(\tau \right)=e^{\nu W(\tau )},$$

with

(29) $$\begin{array}{cc} W(t) & =\langle \phi_{R/L}\left(0,t\right)\phi_{R/L}\left(0,0\right)-\phi_{R/L}^{2}\left(0,0\right)\rangle =\\ & =\ln\left[\frac{{\left|\Gamma \left(1+\frac{\theta }{\omega_{c}}+i\theta t\right)\right|}^{2}}{{\left|\Gamma \left(1+\frac{\theta }{\omega_{c}}\right)\right|}^{2}\left(1+i\omega_{c}t\right)}\right]≃\ln\left[\frac{\pi \theta t}{\sinh\left(\pi \theta t\right)\left(1+i\omega_{c}t\right)}\right]. \end{array}$$

In the above, Equation θ is the temperature ($k_{B}=1$), $\omega_{c}=v/a$ is the high energy cut-off, with *a* the finite length cut-off appearing in Equation (25), and Γ(x) is the Euler’s gamma function. The considered approximation is valid as long as $\omega_{c}\gg \theta$. Let us notice that the expressions are equal for right- and left-movers and, therefore, we have omitted any label *R* or *L*. Moreover, we explicitly used the fact that W(t) is translationally invariant in time.

By inserting these expressions into Equation (21), one finds

(30) $$J_{BS}\left(t\right)=2i\nu e{\left|\lambda \right|}^{2}\int_{0}^{+\infty }d\tau \sin\left[\nu e\int_{t-\tau }^{t}dt^{\prime }V\left(t^{\prime }\right)\right]\left(P_{2\nu }\left(\tau \right)-P_{2\nu }\left(-\tau \right)\right),$$

where we introduced the rescaled tunneling amplitude λ=Λ/(2πa). Notice that the backscattering current satisfies $J_{BS}\left(t\right)=J_{BS}\left(t+T\right)$ as expected due to the periodicity of the voltage drive V(t). It is thus possible to perform an average over one period of the backscattering current thus finding

(31) $$\bar{J_{BS}\left(t\right)}=2i\nu e{\left|\lambda \right|}^{2}\int_{0}^{T}\frac{dt}{T}\int d\tau \sin\left[\nu e\int_{t-\tau }^{t}dt^{\prime }V\left(t^{\prime }\right)\right]P_{2\nu }\left(\tau \right).$$

The presence of a periodic voltage can be conveniently handled by resorting to the photo-assisted coefficients defined by [16,61]

(32) $$p_{l}\left(\alpha \right)=\int_{-T/2}^{T/2}\frac{dt}{T}e^{2i\pi n\frac{t}{T}}e^{-2i\pi \alpha \varphi (t)},$$

with

(33) $$\varphi \left(t\right)=\int_{-\infty }^{t}\frac{dt^{\prime }}{T}{\bar{V}}_{ac}\left(t^{\prime }\right),$$

where ${\bar{V}}_{ac}\left(t\right)$ is the AC part of V(t) with unitary and dimensionless amplitude. Therefore, the backscattering current assumes the final expression

(34) $$\bar{J_{BS}\left(t\right)}=2i\nu e{\left|\lambda \right|}^{2}\sum_{l}{\left|p_{l}\left(\alpha \right)\right|}^{2}\int_{-\infty }^{+\infty }d\tau \sin\left[\left(q+l\right)\omega \tau \right]P_{2\nu }\left(\tau \right).$$

By moving to Fourier space and by introducing the function [58,62,63,64,65]

(35) $${\tilde{P}}_{\nu }\left(E\right)=\int_{-\infty }^{+\infty }dte^{iEt}P_{\nu }\left(t\right)={\left(\frac{2\pi \theta }{\omega_{c}}\right)}^{\nu -1}\frac{e^{E/2\theta }}{\Gamma \left(\nu \right)\omega_{c}}{\left|\Gamma \left(\frac{\nu }{2}-i\frac{E}{2\pi \theta }\right)\right|}^{2},$$

the current in Equation (34) can be recast as

(36) $$\bar{J_{BS}\left(t\right)}=\nu e{\left|\lambda \right|}^{2}\sum_{l}{\left|p_{l}\left(\alpha \right)\right|}^{2}\left\{{\tilde{P}}_{2\nu }\left[\left(q+l\right)\omega \right]-{\tilde{P}}_{2\nu }\left[-\left(q+l\right)\omega \right]\right\}.$$

This final expression explicitly depends on the rescaled amplitudes of both the AC (α) and DC (*q*) contribution to the voltage. Even if frequently assumed equal, these two parameters can be tuned independently [16,53].

## 4. Heat Current

In order to extend the previous analysis to heat transport, we need to properly define the heat current operator. For a system described by a Hamiltonian density H and with particle number density N, the heat density Q is given by

(37) Q=H−μN,

with μ the chemical potential of the lead where the heat density is measured. This definition is motivated by the fact that the energy density depends on an arbitrary energy reference and, thus, it is not a well-defined experimental observable. On the contrary, heat is defined as the energy carried by particles with respect to a given chemical potential, thus motivating the expression in Equation (37).

According to this and considering again the chiral propagation of the bosonic modes along the edges [66], in the chiral Luttinger description (see Equation (1)), one defines the heat densities as

(38) $$Q_{R/L}\left(x,t\right)=\frac{v}{4\pi }{\left(\partial_{x}\Phi_{R/L}\left(x,t\right)\right)}^{2},$$

where all the contributions proportional to the chemical potential $\mu =vk_{F}$ are automatically taken into account [57,67]. Notice that the above equation provides the proper value of the thermal Hall conductance in agreement with the Wiedemann–Franz law [67,68]

The corresponding heat current operators in the terminals 2 and 3 can be expressed in terms of heat density operators [57] as

(39) $$J_{2/3}\left(t\right)=\pm v\;Q_{R/L}\left(\pm d,t\right)$$

due to the chirality of Laughlin edge states.

Proceeding as for the charge current, the heat current operators are represented in powers of the tunneling amplitude Λ,

(40) $$J_{2/3}\left(t\right)=J_{2/3}^{\left(0\right)}\left(t\right)+J_{2/3}^{\left(1\right)}\left(t\right)+J_{2/3}^{\left(2\right)}\left(t\right)+O\left({\left|\Lambda \right|}^{3}\right),$$

with

(41) $$\begin{array}{c} J_{2/3}^{\left(0\right)}\left(t\right)=\pm vQ_{R/L}^{\left(0\right)}\left(\pm d,t\right), \end{array}$$

(42) $$\begin{array}{c} J_{2/3}^{\left(1\right)}\left(t\right)=\pm iv\int_{-\infty }^{t}dt^{\prime }\left[H_{T}\left(t^{\prime }\right),Q_{R/L}^{\left(0\right)}\left(\pm d,t\right)\right], \end{array}$$

(43) $$\begin{array}{c} J_{2/3}^{\left(2\right)}\left(t\right)=\pm i^{2}v\int_{-\infty }^{t}dt^{\prime }\int_{-\infty }^{t^{\prime }}dt^{\prime \prime }\left[H_{T}\left(t^{\prime \prime }\right),\left[H_{t}\left(t^{\prime }\right),Q_{R/L}^{\left(0\right)}\left(\pm d,t\right)\right]\right]. \end{array}$$

In the above equations, we have denoted with $Q_{R/L}^{\left(0\right)}\left(x,t\right)$ the time evolution of heat densities in the absence of tunneling, which can be obtained from the time evolution of bosonic fields in Equation (5) and reads

(44) $$\begin{array}{ccc} Q_{R}^{\left(0\right)}\left(x,t\right) & = & \frac{v}{4\pi }\left[{\left(\partial_{x}\phi_{R}\left(x,t\right)\right)}^{2}+2e\sqrt{\nu }\partial_{x}\phi_{R}\left(x,t\right)V_{R/L}\left(t\mp \frac{x}{v}\right)+\frac{e^{2}\nu }{v}V_{R}^{2}\left(t\mp \frac{x}{v}\right)\right], \end{array}$$

(45) $$\begin{array}{ccc} Q_{L}^{\left(0\right)}\left(x,t\right) & = & \frac{v}{4\pi }{\left(\partial_{x}\phi_{R/L}\left(x,t\right)\right)}^{2}. \end{array}$$

The commutators involving $Q_{R/L}^{\left(0\right)}\left(x,t\right)$ in Equations (42) and (43) are

(46) $$\begin{array}{c} J_{2/3}^{\left(1\right)}\left(t\right)=\pm {\dot{Q}}_{R/L}\left(\pm d,t\right), \end{array}$$

(47) $$\begin{array}{c} J_{2/3}^{\left(2\right)}\left(t\right)=\pm i\int_{-\infty }^{t-\frac{d}{v}}dt^{\prime \prime }\left[H_{t}\left(t^{\prime \prime }\right),{\dot{Q}}_{R/L}\left(\pm d,t\right)\right], \end{array}$$

where

(48) $$\begin{array}{c} {\dot{Q}}_{R}\left(x,t\right)=v\Lambda \left(\partial_{x}+ik_{F}\right)\Psi_{R}^{†}\left(x,t\right)\Psi_{L}\left(x,t\right)+H.c., \end{array}$$

(49) $$\begin{array}{c} {\dot{Q}}_{L}\left(x,t\right)=-v\Lambda \Psi_{R}^{†}\left(x,t\right)\left(\partial_{x}+ik_{F}\right)\Psi_{L}\left(x,t\right)+H.c.. \end{array}$$

According to this, the average of heat current operators in Equation (40) reads

(50) $$\begin{array}{c} \langle J_{2/3}\left(t\right)\rangle =\langle J_{2/3}^{\left(0\right)}\rangle +\langle J_{2/3}^{\left(2\right)}\rangle . \end{array}$$

Analogously with the case of the charge current, one has that $J_{2/3}^{\left(1\right)}$ is zero due to the unbalance between annihilation and creation field operators of each chirality. Focusing on the heat current which is backscattered by the QPC into reservoir 3, one can define the backscattering heat current as

(51) $$J_{BS}\left(t\right)=\langle J_{3}^{\left(2\right)}\left(t\right)\rangle .$$

It can be expressed in terms of Green’s functions in Equations (26) and (27), by exploiting the explicit expression of ${\dot{Q}}_{L}\left(x,t\right)$ in Equations (48) and (49), thus finding

(52) $$\begin{array}{cc} J_{BS}\left(t\right) & ={i|\Lambda |}^{2}\int_{-\infty }^{t-\frac{d}{v}}d\tau [G_{R}^{<}\left(t^{\prime },t-\frac{d}{v}\right)\left(\partial_{t}-ik_{F}v\right)G_{L}^{>}\left(t^{\prime },t-\frac{d}{v}\right)+\\ & +G_{R}^{>}\left(t^{\prime },t-\frac{d}{v}\right)\left(\partial_{t}+ik_{F}v\right)G_{L}^{<}\left(t^{\prime },t-\frac{d}{v}\right)+\\ & -G_{R}^{<}\left(t-\frac{d}{v},t^{\prime }\right)\left(\partial_{t}-ik_{F}v\right)G_{L}^{>}\left(t-\frac{d}{v},t^{\prime }\right)+\\ & -G_{R}^{>}\left(t-\frac{d}{v},t^{\prime }\right)\left(\partial_{t}+ik_{F}v\right)G_{L}^{<}\left(t-\frac{d}{v},t^{\prime }\right)]. \end{array}$$

By recalling the link between Green’s functions and the function $P_{\nu }\left(t\right)$, the heat backscattering current becomes

(53) $$\begin{array}{c} J_{BS}\left(t\right)={i|\lambda |}^{2}\int_{0}^{+\infty }d\tau \cos\left[\nu e\int_{t}^{t-\tau }dt^{\prime \prime }V_{R}\left(t^{\prime \prime }\right)\right]\partial_{\tau }\left[P_{2\nu }\left(\tau \right)-P_{2\nu }\left(-\tau \right)\right], \end{array}$$

which, averaged over one period, reduces to

(54) $$\begin{array}{c} \bar{J_{BS}\left(t\right)}\left(\alpha ,q\right)={\left|\lambda \right|}^{2}\frac{\omega }{2}\sum_{l}{\left|p_{l}\left(\alpha \right)\right|}^{2}\left(q+l\right)\left\{{\tilde{P}}_{2\nu }\left[(q+l)\omega \right]-{\tilde{P}}_{2\nu }\left[-(q+l)\omega \right]\right\}. \end{array}$$

## 5. Mixed Noise and Heat Noise

Concerning the fluctuations, together with the conventional current noise [69], it is possible to define both the autocorrelated mixed noise and heat noise [45,46,50]. For the sake of simplicity, we will focus exclusively on the signal detected in reservoir 3 considering the quantities

(55) $$\begin{array}{c} S_{X}=\int_{0}^{T}\frac{dt}{T}\int_{-\infty }^{+\infty }dt^{\prime }\;\left[\langle J_{3}\left(t^{\prime }\right)J_{3}\left(t\right)\rangle -\langle J_{3}\left(t^{\prime }\right)\rangle \;\langle J_{3}\left(t\right)\rangle \right]\;, \end{array}$$

(56) $$\begin{array}{c} S_{Q}=\int_{0}^{T}\frac{dt}{T}\int_{-\infty }^{+\infty }dt^{\prime }\;\left[\langle J_{3}\left(t^{\prime }\right)J_{3}\left(t\right)\rangle -\langle J_{3}\left(t^{\prime }\right)\rangle \;\langle J_{3}\left(t\right)\rangle \right].\; \end{array}$$

Notice that analogous expressions can be derived focusing on the reservoir 2.

The perturbative expansion of charge and heat current operators in Equations (13) and (40) allows for also expressing these quantities perturbatively in Λ, namely

(57) $$\begin{array}{c} S_{X}=S_{X}^{\left(02\right)}+S_{X}^{\left(20\right)}+S_{X}^{\left(11\right)}+O\left({\left|\Lambda \right|}^{3}\right), \end{array}$$

(58) $$\begin{array}{c} S_{Q}=S_{Q}^{\left(02\right)}+S_{Q}^{\left(20\right)}+S_{Q}^{\left(11\right)}+O\left({\left|\Lambda \right|}^{3}\right), \end{array}$$

where

(59) $$\begin{array}{c} S_{X}^{\left(ij\right)}=\int_{0}^{T}\frac{dt}{T}\int_{-\infty }^{+\infty }dt^{\prime }\left\{\langle J_{3}^{\left(i\right)}\left(t^{\prime }\right)J_{3}^{\left(j\right)}\left(t\right)\rangle -\langle J_{3}^{\left(i\right)}\left(t^{\prime }\right)\rangle \langle J_{3}^{\left(j\right)}\left(t\right)\rangle \right\}, \end{array}$$

(60) $$\begin{array}{c} S_{Q}^{\left(ij\right)}=\int_{0}^{T}\frac{dt}{T}\int_{-\infty }^{+\infty }dt^{\prime }\left\{\langle J_{3}^{\left(i\right)}\left(t^{\prime }\right)J_{3}^{\left(j\right)}\left(t\right)\rangle -\langle J_{3}^{\left(i\right)}\left(t^{\prime }\right)\rangle \langle J_{3}^{\left(j\right)}\left(t\right)\rangle \right\}. \end{array}$$

In the perturbative expansions of Equations (57) and (58), the only surviving terms are $S_{X}^{\left(11\right)}$ and $S_{Q}^{\left(11\right)}$, since one can show that [50,66]

(61) $$\begin{array}{c} S_{X}^{\left(02\right)}=S_{X}^{\left(20\right)}=0, \end{array}$$

(62) $$\begin{array}{c} S_{Q}^{\left(02\right)}=S_{Q}^{\left(20\right)}=0. \end{array}$$

Mixed and heat noises are obtained in terms of Green’s functions as

(63) $$\begin{array}{c} S_{X}=i{\left|\Lambda \right|}^{2}\int_{0}^{T}\frac{dt}{T}\int_{-\infty }^{+\infty }dt^{\prime }\left[G_{R}^{<}\left(t^{\prime },t\right)\left(\partial_{t}-ik_{F}v\right)G_{L}^{<}\left(t^{\prime },t\right)-G_{R}^{<}\left(t^{\prime },t\right)\left(\partial_{t}+ik_{F}v\right)G_{L}^{<}\left(t^{\prime },t\right)\right], \end{array}$$

(64) $$\begin{array}{cc} S_{Q} & ={\left|\Lambda \right|}^{2}\int_{0}^{T}\frac{dt}{T}\int_{-\infty }^{+\infty }dt^{\prime }[G_{R}^{<}\left(t^{\prime },t\right)\left(\partial_{t^{\prime }}+ik_{F}v\right)\left(\partial_{t}-ik_{F}v\right)G_{L}^{<}\left(t^{\prime },t\right)+\\ & +G_{R}^{<}\left(t^{\prime },t\right)\left(\partial_{t^{\prime }}-ik_{F}v\right)\left(\partial_{t}+ik_{F}v\right)G_{L}^{<}\left(t^{\prime },t\right)] \end{array}$$

and, in terms of the function $P_{\nu }\left(t\right)$ in Equation (28), they can be rewritten as

(65) $$\begin{array}{c} S_{X}={2\nu e|\lambda |}^{2}\int_{0}^{T}\frac{dt}{T}\int_{-\infty }^{+\infty }dt^{\prime }\sin\left[\nu e\int_{t^{\prime }}^{t}dt^{\prime \prime }V\left(t^{\prime \prime }\right)\right]P_{\nu }\left(t^{\prime }-t\right)\partial_{t^{\prime }}P_{\nu }\left(t^{\prime }-t\right), \end{array}$$

(66) $$\begin{array}{c} S_{Q}={2|\lambda |}^{2}\int_{0}^{T}\frac{dt}{T}\int_{-\infty }^{+\infty }dt^{\prime }\cos\left[\nu e\int_{t^{\prime }}^{t}dt^{\prime \prime }V\left(t^{\prime \prime }\right)\right]P_{\nu }\left(t^{\prime }-t\right)\partial_{t}\partial_{t^{\prime }}P_{\nu }(t^{\prime }-t). \end{array}$$

Using again the series expansion of the voltage dependent phase factor and the Fourier transform for $P_{\nu }\left(t^{\prime }-t\right),$ one is left with

(67) $$\begin{array}{c} S_{X}\left(\alpha ,q\right)=\frac{\nu e\omega }{2}{\left|\lambda \right|}^{2}\sum_{l}{\left|p_{l}\left(\alpha \right)\right|}^{2}\left(q+l\right)\left\{{\tilde{P}}_{2\nu }\left[(q+l)\omega \right]+{\tilde{P}}_{2\nu }\left[-(q+l)\omega \right]\right\}, \end{array}$$

(68) $$\begin{array}{c} S_{Q}\left(\alpha ,q\right)=\frac{{\left|\lambda \right|}^{2}}{2\pi }\sum_{l}{\left|p_{l}\left(\alpha \right)\right|}^{2}\int_{-\infty }^{+\infty }dEE^{2}{\tilde{P}}_{\nu }\left(E\right)\left\{{\tilde{P}}_{\nu }\left[(q+l)\omega -E\right]+{\tilde{P}}_{\nu }\left[-(q+l)\omega -E\right]\right\}, \end{array}$$

where we have explicitly indicated the dependence on the AC (α) and DC (*q*) voltage amplitude.

To perform the integral in the equation for $S_{Q}$, we exploit the identity

(69) $$\begin{array}{c} \int_{-\infty }^{+\infty }\frac{dY}{2\pi }Y^{2}{\tilde{P}}_{g_{1}}\left(Y\right){\tilde{P}}_{g_{2}}\left(X-Y\right)=\frac{{\tilde{P}}_{g_{1}+g_{2}}\left(X\right)}{1+g_{1}+g_{2}}\left[g_{1}g_{2}\pi^{2}\theta^{2}+\frac{g_{1}\left(1+g_{1}\right)}{g_{1}+g_{2}}\omega^{2}\right] \end{array}$$

obtaining the expression

(70) $$\begin{array}{c} S_{Q}\left(\alpha ,q\right)={\left|\lambda \right|}^{2}\sum_{l}{\left|p_{l}\left(\alpha \right)\right|}^{2}\left[\frac{2\pi^{2}\nu^{2}}{1+2\nu }\theta^{2}+\frac{1+\nu }{1+2\nu }{\left(q+l\right)}^{2}\omega^{2}\right]\left\{{\tilde{P}}_{2\nu }\left[(q+l)\omega \right]+{\tilde{P}}_{2\nu }\left[-(q+l)\omega \right]\right\}. \end{array}$$

At temperature zero, the above expressions reduce to

(71) $$\begin{array}{c} S_{X}\left(\alpha ,q\right)={\nu e|\lambda |}^{2}\frac{\pi }{\Gamma (2\nu )}{\left(\frac{\omega }{\omega_{c}}\right)}^{2\nu }\sum_{l}|p_{l}{\left(\alpha \right)|}^{2}{\left|q+l\right|}^{2\nu }\mathrm{sign}\left(q+l\right), \end{array}$$

(72) $$\begin{array}{c} S_{Q}\left(\alpha ,q\right)={\omega |\lambda |}^{2}\frac{\pi (1+\nu )}{\Gamma (2\nu )(1+2\nu )}{\left(\frac{\omega }{\omega_{c}}\right)}^{2\nu }\sum_{l}|p_{l}{\left(\alpha \right)|}^{2}{\left|q+l\right|}^{2\nu +1}. \end{array}$$

Here, the chiral free fermion case is recovered directly by inserting ν=1. It is worth noticing that this peculiar state can be described exactly (to all order in the tunneling amplitude) in terms of the scattering theory [69,70]. Moreover, at this value of the filling factor, the dependence on *a* (finite length cut-off) disappears.

These noises will be investigated in the following with the aim of carrying out their spectroscopic analysis. For sake of simplicity, we will focused on the zero temperature limit, the finite temperature correction being negligible as far as θ≪ω.

## 6. Results

### 6.1. Heat Current

**Figure 2:**
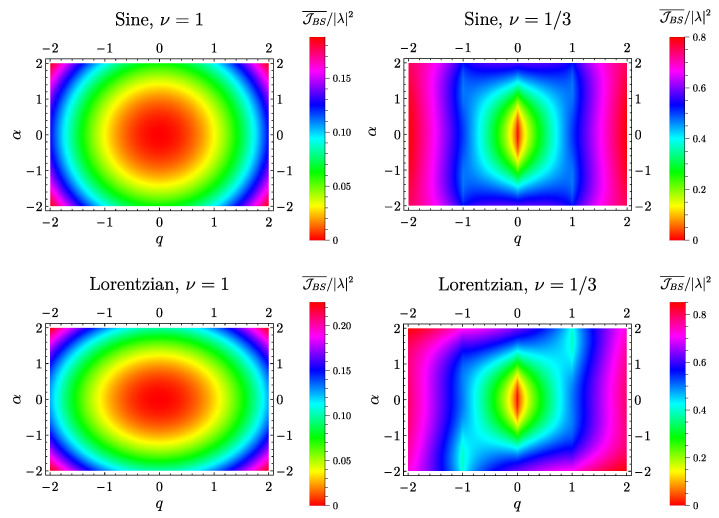
Density plot of the averaged heat current $\bar{J_{BS}}$ (in units of ${\left|\lambda \right|}^{2}$) as a function of the DC voltage amplitude *q* ($\hat{x}$-axis) and the AC voltage amplitude α ($\hat{y}$-axis). Other parameters are: η=0.1, θ=0 and $\omega_{c}=10\omega$.

The behavior of the photo-assisted charge current as a function of the AC and DC voltage contribution has been already discussed both in the non-interacting [16] and in the strongly interacting regime [17,53], the latter case showing divergencies at zero temperature signature of the limitations of the perturbative approach in this regime. In Figure 2, we report the density plots of its heat counterpart whose functional form has been derived in Equation (54). The first row represents the case of a sinusoidal drive with

(73) $$V_{ac}^{\left(\cos\right)}\left(t\right)=-\frac{\omega \alpha }{e}\cos\left(\omega t\right)$$

both in the free fermion case ν=1 (top left panel) and at ν=1/3 (top right panel). Here, all curves are mirror symmetric with respect to α=0 ($\bar{J_{BS}}\left(\alpha ,q\right)=\bar{J_{BS}}\left(-\alpha ,q\right)$) and q=0 ($\bar{J_{BS}}\left(\alpha ,q\right)=\bar{J_{BS}}\left(\alpha ,-q\right)$) and consequently satisfy

(74) $$\bar{J_{BS}}\left(\alpha ,q\right)=\bar{J_{BS}}\left(-\alpha ,-q\right).$$

Both of these remarkable features are a direct consequence of the symmetries of the photo-assisted tunneling amplitudes. Indeed, for this kind of drive, one has [16,61]

(75) $$p_{l}^{\left(\cos\right)}\left(\alpha \right)=J_{l}\left(-\alpha \right),$$

with $J_{l}$ Bessel function of order *l*. These amplitudes satisfy

(76) $$p_{l}^{\left(\cos\right)}\left(\alpha \right)=p_{l}^{\left(\cos\right)}\left(-\alpha \right)$$

and

(77) $$p_{l}^{\left(\cos\right)}\left(\alpha \right)=p_{-l}^{\left(\cos\right)}\left(\alpha \right).$$

Considering the case of a periodic train of Lorentzian pulses of the form

(78) $$V_{ac}^{\left(\mathrm{Lor}\right)}\left(t\right)=\frac{\omega }{\pi e}\sum_{l=-\infty }^{+\infty }\frac{\eta }{\eta^{2}+{\left(\frac{t}{T}-l\right)}^{2}}-\frac{\omega \alpha }{e},$$

with width at half height given by η and where the photo-assisted tunneling amplitudes are given by

(79) $$p_{l}^{\left(\mathrm{Lor}\right)}\left(\alpha \right)=\alpha \sum_{s=0}^{+\infty }\frac{\Gamma (\alpha +l+s)}{\Gamma (\alpha +1-s)}\frac{{\left(-1\right)}^{s}e^{-2\pi \eta (2s+l)}}{\Gamma (l+s+1)\Gamma (s+1)}.$$

In this case, one observes that, at ν=1 (bottom left panel of Figure 2), the heat current is still highly symmetric as in the case of the sinusoidal drive. This is an accidental consequence of the fact that, at this value of the filling factor for every periodic voltage drive, one has [50]

(80) $$\bar{J_{BS}}\left(\alpha ,q\right)\propto \sum_{l=-\infty }^{+\infty }{\left|p_{l}\left(\alpha \right)\right|}^{2}{\left(q+l\right)}^{2}=\frac{e}{\hbar \omega }\int_{0}^{T}\frac{dt}{T}V^{2}\left(t\right)=\frac{e}{\hbar \omega }V_{dc}^{2}+\frac{e}{\hbar \omega }\int_{0}^{T}\frac{dt}{T}V_{ac}^{2}\left(t\right),$$

which is manifestly insensitive to the overall sign of both the DC and the AC contribution to the voltage. This is no more true for what concerns the filling factors in the Laughlin sequence (bottom right panel of Figure 2) where

(81) $$\begin{array}{ccc} \bar{J_{BS}}\left(\alpha ,q\right) & \neq & \bar{J_{BS}}\left(-\alpha ,q\right), \end{array}$$

(82) $$\begin{array}{ccc} \bar{J_{BS}}\left(\alpha ,q\right) & \neq & \bar{J_{BS}}\left(\alpha ,-q\right). \end{array}$$

However, the condition

(83) $$p_{l}^{\left(\mathrm{Lor}\right)}\left(\alpha \right)=p_{-l}^{\left(\mathrm{Lor}\right)}\left(-\alpha \right)$$

leads to the residual symmetry

(84) $$\bar{J_{BS}}\left(\alpha ,q\right)=\bar{J_{BS}}\left(-\alpha ,-q\right).$$

### 6.2. Mixed Noise

**Figure 3:**
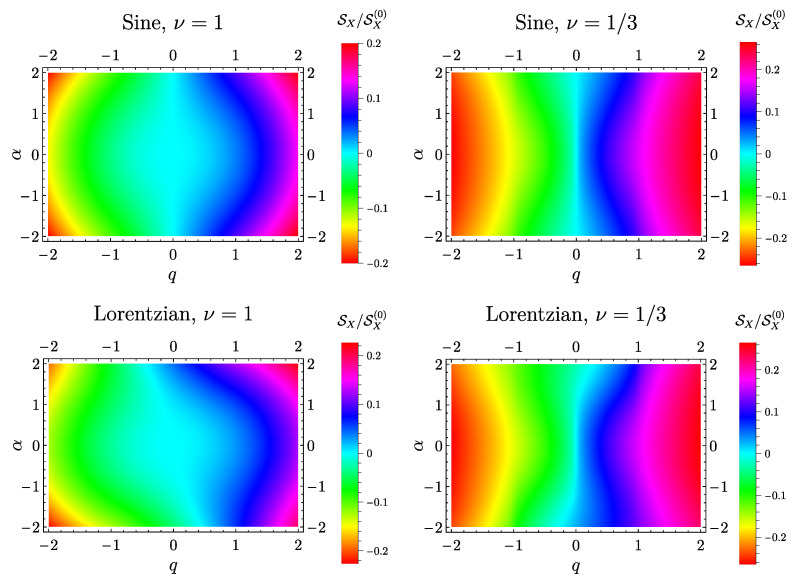
Density plot of the mixed noise $S_{X}$ (in units of $S_{X}^{\left(0\right)}=e{\left|\lambda \right|}^{2}$) as a function of the DC voltage amplitude *q* ($\hat{x}$-axis) and the AC voltage amplitude α ($\hat{y}$-axis). Other parameters are: η=0.1, θ=0 and $\omega_{c}=10\omega$.

Density plots in Figure 3 show the behavior of the charge-heat mixed noise as a function of the AC voltage amplitude α and of the DC voltage amplitude *q*. In particular, the first row represents the case of a sinusoidal drive both in the free fermion case ν=1 (top left panel) and at ν=1/3 (top right panel). In this case, all curves show the properties $S_{X}\left(\alpha ,q\right)=S_{X}\left(-\alpha ,q\right)$, $S_{X}\left(\alpha ,q\right)=-S_{X}\left(\alpha ,-q\right)$ and consequently

(85) $$S_{X}\left(\alpha ,q\right)=-S_{X}\left(-\alpha ,-q\right).$$

The curves at ν=1 are increasing (decreasing) for increasing |α| at positive (negative) *q*, while the opposite is true for ν=1/3. This overall profile is dictated by the power-law behavior (exponent 2ν in Equation (71)) which is parabolic for ν=1 and sub-linear (2ν=2/3, further suppressed by the fast decreasing photo-assisted tunneling amplitude) for ν=1/3.

While these general considerations about the asymptotic behavior of the curves at increasing |α| still hold in the case of the Lorentzian case (second row of Figure 3), here both plots are manifestly asymmetric. Again, this fact can be directly attributed to the (lack of) symmetries of the photo-assisted tunneling amplitudes $p_{l}^{\left(\mathrm{Lor}\right)}\left(\alpha \right)$. This asymmetry in α is very evident at small values of |q| and becomes progressively less important by increasing |q|. Despite this, it is possible to note that Equation (85) is still satisfied.

### 6.3. Heat Noise

**Figure 4:**
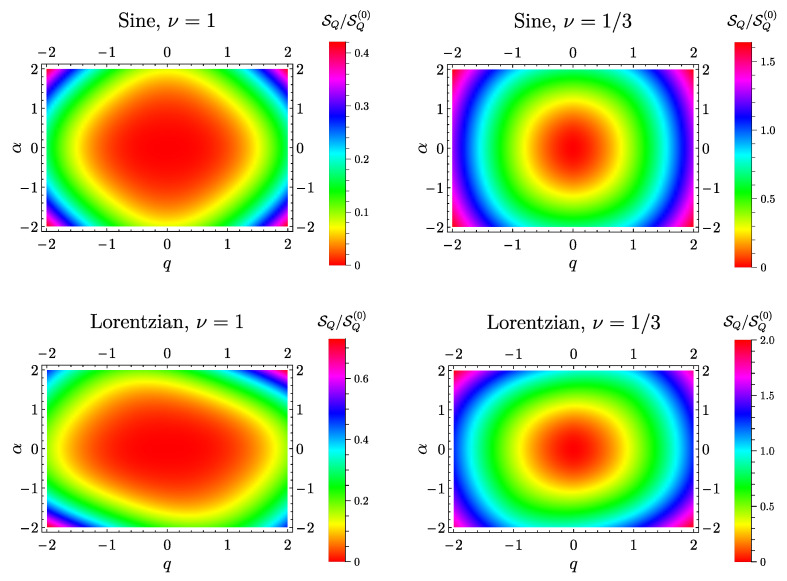
Density plot of the heat noise $S_{Q}$ (in units of $S_{Q}^{\left(0\right)}=\omega {\left|\lambda \right|}^{2}$) as a function of DC voltage amplitude *q* ($\hat{x}$-axis) and the AC voltage amplitude α ($\hat{y}$-axis). Other parameters are: η=0.1, θ=0 and $\omega_{c}=10\omega$.

The density plots of the heat current fluctuations as a function of the AC voltage amplitude α and of the DC voltage amplitude *q* are reported in Figure 4. As before, the two upper panels show the sinusoidal drive case both in the free fermion case ν=1 (top left panel) and at ν=1/3 (top right panel). Here, according to the conditions in Equations (76) and (77), all curves satisfy $S_{Q}\left(\alpha ,q\right)=S_{Q}\left(-\alpha ,q\right)$. In addition, differently from what happens in the mixed noise case, one has that this quantity is positively defined and that $S_{Q}\left(\alpha ,q\right)=S_{Q}\left(\alpha ,-q\right)$.

Because of the greater power-law in Equation (72) with respect to the one in Equation (71), the curves are always increasing by increasing |α| for both the free and the strongly interacting case. The same is true also for the Lorentzian drive (bottom panels of Figure 4) although, in this case, the asymmetry of the $p_{l}^{\left(\mathrm{Lor}\right)}\left(\alpha \right)$ directly reflects in the asymmetry of the curves for α→−α ($S_{Q}\left(\alpha ,q\right)\neq S_{Q}\left(-\alpha ,q\right)$) and q→−q ($S_{Q}\left(\alpha ,q\right)\neq S_{Q}\left(\alpha ,-q\right)$). However, the curves are characterized by the condition

(86) $$S_{Q}\left(\alpha ,q\right)=S_{Q}\left(-\alpha ,-q\right)$$

due to the property in Equation (83).

## 7. Conclusions

In this work, we investigated charge-heat mixed noise and heat noise generated by a periodic time-dependent voltage in a quantum point contact geometry implemented in a quantum Hall bar. We focused on the weak backscattering regime of the tunneling barrier and we evaluated fluctuations of charge and heat currents at lowest order in tunneling the amplitude. We provided a spectroscopic analysis of these quantities by tuning independently the AC and DC amplitudes of the voltage drive for integer and fractional filling factors. By focusing on a cosine drive and a periodic train of Lorentzian pulse, we proved that both charge-heat noise and heat noise reveal the symmetry properties of the photo-assisted coefficients of the applied drive. The different power-laws that govern the behavior of mixed and heat noises give rise to two distinct profiles when these quantities are plotted as a function of the AC amplitude. In particular, charge-heat noise displays an opposite monotonicity for the case of integer and fractional filling factors, thus revealing the presence of a sub-linear power-law decay typical of Luttinger liquid physics.

## Abbreviations

The following abbreviations are used in this manuscript:

FQH	Fractional Quantum Hall
QPC	Quantum Point Contact
EQO	Electron Quantum Optics
